# Profile of Drug Utilization in Patients with Rare Diseases in Tuscany, Italy: A Population-Based Study

**DOI:** 10.3390/ijerph20020937

**Published:** 2023-01-04

**Authors:** Francesca Gorini, Michele Santoro, Anna Pierini, Lorena Mezzasalma, Silvia Baldacci, Alessio Coi

**Affiliations:** 1 Institute of Clinical Physiology, National Research Council, 56124 Pisa, Italy; 2 Fondazione Gabriele Monasterio CNR-Regione Toscana, 56124 Pisa, Italy

**Keywords:** rare disease, drug, prevalence of use, intensity of use, consumption, population-based

## Abstract

Patients with rare diseases (RDs) generally have delayed diagnosis and misdiagnosis, which lead to inappropriate care or the need to modify treatment during the course of the disease. The medical care of RD patients can be further complicated by the presence of comorbidities. In this population-based study, we evaluated the prevalence, intensity of use, and consumption of drugs prescribed to RD patients residing in Tuscany (Italy) in the years 2008–2018. Data from the Registry of Rare Diseases of Tuscany were integrated with information retrieved from regional pharmaceutical prescription databases. The overall prevalence of drug use in the RD patients was 85.4%. Drugs for the alimentary tract and metabolism and antiinfectives for systemic use showed the highest prevalence of use, while drugs for the nervous system had the highest intensity of use only in the pediatric patients. The adults exhibited a female preponderance in terms of the prevalence of use and drug consumption in almost all the age groups and therapeutic categories. Conversely, a higher prevalence of use was observed in the male children. These results provide relevant insights into drug profiles in RD patients, representing a first step for future analyses to monitor changes in drug utilization in patients with RDs over time.

## 1. Introduction

The European Union (EU) defines a disease as rare when it affects fewer than 1 in 2000 people [1]: however, these conditions are defined differently according to the specific jurisdiction responsible for their definition [2].

Currently, around 7000 known rare diseases (RDs) have been identified, affecting more than 300 million people worldwide, including 30 million in the EU [3,4,5]. Hence, although, individually, most RDs might affect only a few hundred to a few thousand people, it is estimated that 6% of the world’s population suffers from a RD [3,6].

While it has been widely recognized that approximately 80% of RDs have a genetic etiology [2], patients with RDs have generally delayed diagnosis and misdiagnosis, mainly due to variable presentations, which involve clinical signs and symptoms that are similar to those of other more common disorders [7,8]. The misdiagnosis and lack of diagnosis of RDs can lead to inappropriate care or the need to modify treatment during the course of the disease [6]. Furthermore, fewer than 6% of RDs have specific pharmacological treatments approved, termed as orphan drugs, due both to the difficulties in designing clinical trials large enough to determine the benefits and adverse effects and to the high costs, which can make the treatments inconvenient [5,9].

In Italy, it is estimated that there are 3 million cases of patients with RDs [10]. Most RDs are serious or life-threatening conditions characterized by substantial morbidity and early mortality and, consequently, are associated with a significant economic burden on individuals and society [2,6,11]. Beyond the limited availability of specific therapies for rare disorders, the medical care of RD patients can be further complicated by the presence of comorbidities that require additional treatment, increasing the overall health burden of RDs [12,13,14]. Altogether, RDs therefore represent a relevant social and public health issue due, in particular, to the direct medical costs associated with their diagnosis and treatment [15].

In this population-based study, we analyzed the prevalence, intensity of use and consumption of drugs prescribed and reimbursed by the national healthcare system for RD patients residing in Tuscany (Italy). A multi-source approach was applied, and data from the Registry of Rare Diseases of Tuscany (RRDT) were integrated with information collected in the regional pharmaceutical prescription databases.

## 2. Materials and Methods

### 2.1. Study Design, Data Source and Study Subjects

In this retrospective cohort study, the study period covered the years 2008–2018. The monitored population included all subjects residing in Tuscany, an Italian region of 3,701,343 inhabitants (source: Italian National Institute of Statistics as of 1 January 2018), and who had been diagnosed between 1 January 2000 and 31 December 2017 with one of the RDs surveilled by the population-based RRDT. The registry, active since 2005 and based on a regional network of health centers, includes all RDs reported on the list of the Italian law (Decree of the President of the Council of Ministers, 01/2017) (Table S1), which are identified by a specific six-digit code for co-payment exemptions.

Drug prescriptions were recovered from two regional pharmaceutical administrative databases collecting information on all drugs reimbursed by the Italian healthcare system and dispensed in community (i.e., public and private pharmacies) and hospital pharmacies (i.e., medications directly supplied by hospitals) for outpatient use.

### 2.2. Study Outcomes and Data Analysis

Drugs were coded using Anatomical Therapeutic Chemical (ATC) classification and therefore divided into fourteen anatomical or pharmacological groups (1st level) (Table 1).

Considering all RDs, the population study was stratified into two age groups: adult (age ≥ 18 years) and pediatric (age < 18 years). The prevalent cases on 1 January of each year of the study period were calculated. All RD patients were pseudonymized by a unique identification number and then linked to the regional pharmaceutical databases.

For pediatric and adult patients, the drug-use profile was assessed through three indicators: (1) the prevalence of use, a measure of exposure, was evaluated per calendar year and calculated as the ratio between the number of cases with at least one pharmaceutical prescription and the number of prevalent cases in the same year. The prevalence rates were expressed as percentage and stratified by year, age group, therapeutic category and sex; (2) the prescriptions/users (Pr/Us) ratio, a measure of intensity of use, was calculated by dividing the total number of prescriptions of drugs belonging to each therapeutic category by the number of users (cases with at least one prescription) in the same therapeutic category and in the same year [16,17]; (3) the total defined daily dose (DDD, the assumed average maintenance dose per day for a drug used as per its main indication in adults) utilized, on average, on any given day of the year and analyzed in a group of 1000 cases (DDD/1000 cases per day) [18]. The DDDs were not calculated for the following categories/subgroups, according to the guidelines (https://www.whocc.no/atc_ddd_index/, accessed on 15 October 2022): A15—appetite stimulants; B05—blood substitutes and perfusion solutions; C05—vasoprotectives; D—dermatologicals; J06—immune sera and immunoglobulins (except for nebacumab in J06BC01); J07—vaccines; L01—antineoplastic agents (except for the protein kinase inhibitors in L01E only); P03—ectoparasiticides, incl. scabicides, insecticides and repellents; S02—otologicals; S03—ophthalmological and otological preparations; V09—diagnostic radiopharmaceuticals; V10—therapeutic radiopharmaceuticals. Furthermore, since the population analyzed comprised patients with RDs, the subgroup N07B—drugs used in addictive disorders—was also not included in the estimate of DDD. The indicator DDD/1000 cases per day was stratified by age class, sex and calendar year.

The proportion test was used to explore differences by gender about percentages in the study cohort, and a *p*-value < 0.05 was considered statistically significant. The data were analyzed with Stata, version 16 [19].

## 3. Results

### 3.1. Prevalence of Use

**Figure 1:**
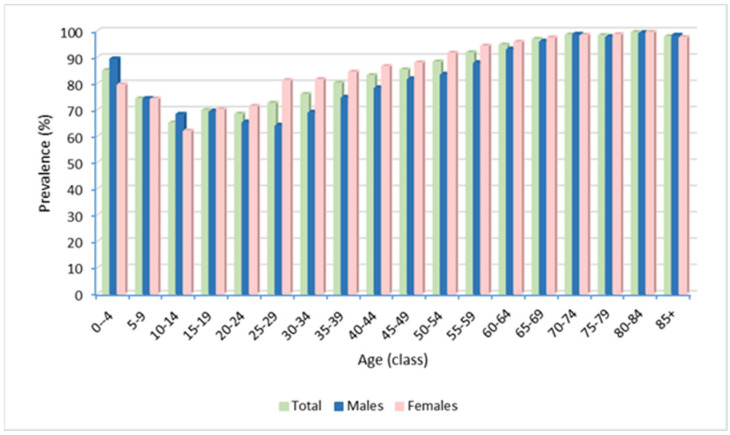
Prevalence of use of all therapeutic categories by age and sex in 2018.

The estimated overall prevalence of use in the last year of the study period (2018) was 85.4%. In the analysis of prevalence by age and sex for all therapeutic categories in the same year, 85% of infants up to 4 years were prescribed at least one medication (Figure 1). We observed a progressive decrease in the subsequent age groups, until the age of 20, followed by an increase with ageing of patients (reaching 99.4% between 80–84 years of age).

Overall, the prevalence was significantly higher in females than males (87.7% vs. 82.6%, *p* < 0.0001), and in adults (84.7% vs. 77.2%, *p* < 0.0001), including those aged 20–69 years (89.2% vs. 81.4%, *p* < 0.0001). Conversely, a higher prevalence rate was found in males (74.8 vs. 69.4%, *p* = 0.001) in the age group 0–14 years (Figure 1).

**Figure 2:**
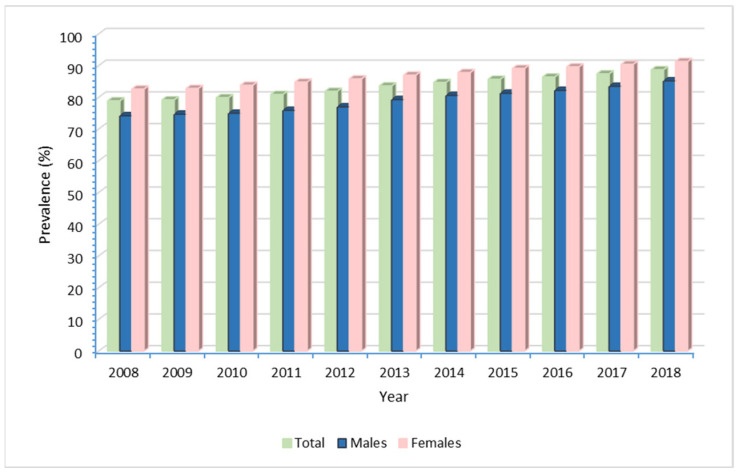
Prevalence of use in adult RD patients (≥18 years) by calendar year.

The proportion of adult users varied between 78.9% in 2008 and 88.7% in 2018, with a significantly higher prevalence among females (*p* < 0.0001) throughout the entire study period (Figure 2).

**Figure 3:**
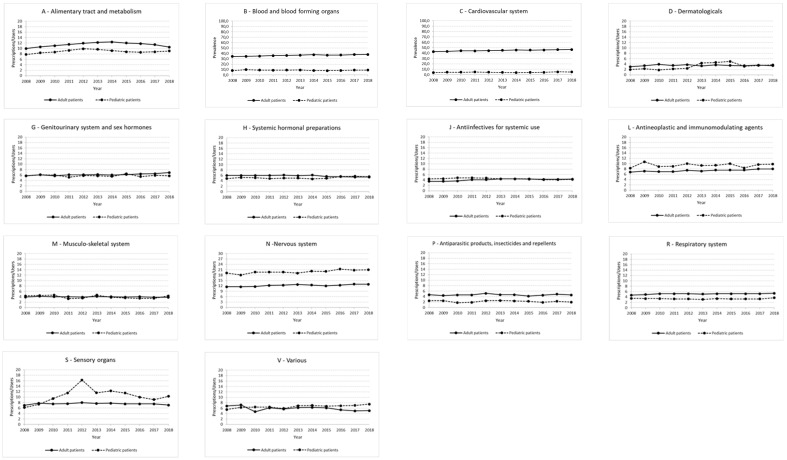
Graphs showing the prevalence of use for each therapeutic category in adult and pediatric RD patients in the period 2008–2018.

Among the adult RD patients, the therapeutic categories with the highest prevalence of use were Group A (alimentary tract and metabolism), with values ranging from 53.8% in 2008 to 61.2% in 2018 and Group J (antiinfectives for systemic use), characterized by values between 55.9–59.1% over the study period (Figure 3). Group M (musculo-skeletal system) was instead characterized by a slight decrease in the prevalence of use (from 38.3% in 2008 to 31.7% in 2018). Group H (systemic hormonal preparations, excluding sex hormones and insulin), Group N (nervous system) and Group R (respiratory system) had approximately steady trends (ranges between 39.8–41.7%, 38.8–41.8%, and 22.2–24.2%, respectively). On the other hand, the therapeutic groups with the lowest percentages of users were: Group D (dermatologicals), Group P (antiparasitic products, insecticides and repellents, with antimalarials representing 77.9% of prescription prevalence in this category), and Group V (various; users-of-medical-gases subgroup accounted for 54.8% of total users in this category in 2018), whose values increased from 0.8% in 2008 to 3.6% in 2015 (Figure 3).

Additionally, for Groups A, H, J, M, N, P, and L (antineoplastic and immunomodulating agents), the prevalence of use among females was significantly higher than among males in each year of the study period (*p* < 0.0001). For Group B (blood and blood-forming organs) a female preponderance (*p* < 0.0001) was found in all the years except in 2008. By contrast, for Group G—genitourinary system and sex hormones—the prevalence was significantly higher in males throughout the study period (*p* < 0.0001) (data not shown).

Of the pediatric RD patients, 71.8% of the subjects received at least one drug prescription in 2018. The analysis by age class revealed a value of 85.0% in the age group 0–4 years, with a peak in infants under the age of 1 year. In the same year, the prevalence of use of all therapeutic categories was higher in males than in females (73.8% vs. 69.5%, *p* = 0.018), and significant sex differences in drug exposure were also detected in the 0–4-year class (89.3% in males vs. 79.6% in females, *p* = 0.002) and in the 10–14-year class (68.3% in males vs. 62.0% in females, *p* = 0.021). When evaluating the entire study period, the highest prevalence of use in pediatric patients was observed in Group J, albeit with a decreasing trend (with the proportion of patients with at least one prescription in a year ranging from 63.0% in 2008 to 48.2% in 2018), followed by Group A, showing an increasing trend (range from 12.9% in 2008 to 23.4% in 2018, with a maximum of 25.3% in 2016). The lowest prescription prevalence was seen in Groups D, G (genitourinary system and sex hormones), M and P, all showing a steady trend with values, between 0.9–1.7%, 1.1–1.7%, 2.4–3.3% and 1.4–2.4%, respectively. By contrast, Group S (sensory organs) was characterized by a decreasing trend (from 1.7% in 2008 to 0.7% in 2017) (Figure 3).

Considering the groups with the highest prevalence of use in pediatric patients in the last year of the study period (2018), amoxicillin/clavulanic acid was the most prescribed substance (31.2% of prevalence) in Group R, followed by cefixime (9.4% of prevalence). Within Groups A, H, R and N, cholecalciferol, betamethasone, salbutamol and valproic acid were the active ingredients with the greatest prevalence of use (12.0%, 12.5%, 8.2% and 5.5%, respectively). Valproic acid was the substance with the highest consumption (1003 prescriptions per 1000 cases), followed by amoxicillin/clavulanic acid and somatropin (715 and 583 prescriptions per 1000 cases, respectively) (Table 2).

The analysis by sex revealed a significantly higher prevalence of use among males for amoxicillin/clavulanic acid alone (*p* = 0.038) in Group J; for most anti-asthma drugs (i.e., salbutamol, beclomethasone and fluticasone) in Group R, with males exposed 11.6% to 15.5% more than females (*p* ≤ 0.034 for all three substances and *p* ≤ 0.0001 considering the whole group); and both for the whole category (*p* = 0.045) and for betamethasone, for which the male to female prevalence ratio was 1.26 (*p* = 0.011) in Group H. A significant increase in exposure to medications in Group J and Group R was detected in male infants aged 0–4 years (*p* = 0.012 and *p* = 0.027, respectively; data not shown).

### 3.2. Intensity of Use

**Figure 4:**
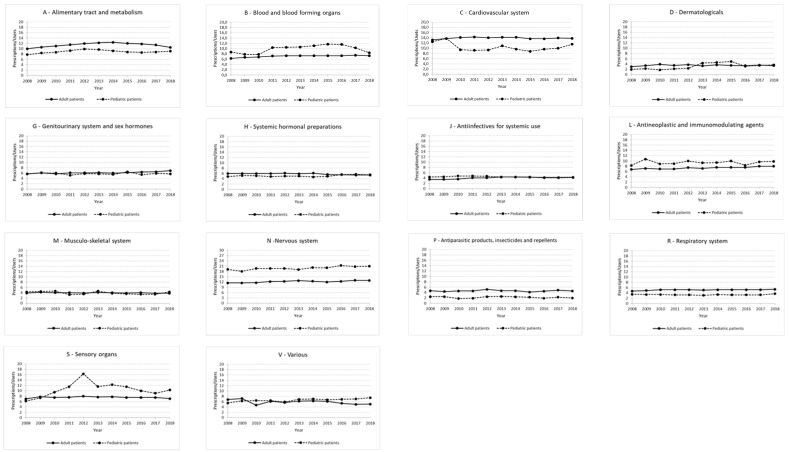
Graphs showing the intensity of use (expressed by the ratio prescriptions/users) for each therapeutic category in adult and pediatric RD patients in the period 2008–2018.

Groups C, N and A, with ranges of 13.2–14.3, 11.5–13.0 and 10.0–12.4 Pr/Us, respectively, displayed the highest intensity of use in the adult RD patients (Figure 3). Lower values of intensity of use (on average around 7–7.5 Pr/Us) were shown by Groups B, L and Group J, with a range of 3.5–4.4 Pr/Us; Group M, with values of 3.8–4.2 Pr/Us and Group D, with a range varying from 3.0 to 3.9 Pr/Us, were characterized by the lowest intensity of use in our cohort (Figure 4).

In the pediatric RD patients, the highest intensity of use was observed in Group N, with a range of values between 18.1 and 21.4 Pr/Us. The lowest intensity of use was reported for Group D, varying from 1.8 to 5.0 Pr/Us and Group P (with antinematodal agents representing 32.2% of the prescriptions in this category in 2018), which showed a range of 1.8–2.6 Pr/Us (Figure 4).

### 3.3. Drug Consumption

As observed for the prevalence of use, the drug consumption was characterized by a peak between 0 and 4 years of age (5331 DDD/1000 cases/day) and then decreased up to 19 years of age. Limiting the analysis to the age group of 0*–*1 years, a consumption of 6549.9 DDD/1000 cases/day was observed.

**Figure 5:**
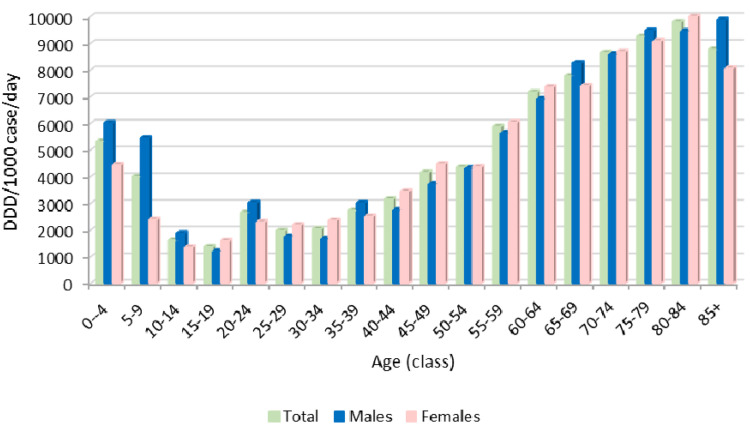
Pharmaceutical consumption (expressed as DDD/1000 cases/day) for all therapeutic categories by age and sex, in 2018.

In adults with RDs, 2662.2 DDD/1000 cases/day were registered between the ages of 20*–*24 and, after a slight decrease, the drug consumption reached 9813.8 DDD/1000 cases/day in patients between the ages of 80*–*85, after which it declined again in the subjects older than 85 years of age (8773 DDD/1000 cases/day). Furthermore, higher consumption in males was observed in pediatric RD patients up to 14 years of age, especially in infants under 4 years of age and, among adults, in a few age groups, particularly in the elderly over 85 years of age (Figure 5).

**Figure 6:**
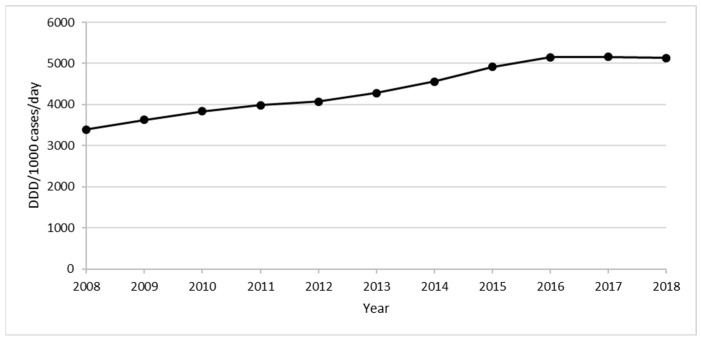
Consumption of all drugs in the period 2008–2018.

Considering the entire study period, the drug consumption registered a progressive increase from 3396.6 DDD/1000 cases per day in 2008 to 5152.5 DDD/1000 cases per day in 2016, followed by a steady profile in the last two years (5199.8 DDD/1000 cases per day in 2018) (Figure 6).

## 4. Discussion

In this study, we found that in 2018, the overall prevalence of drug use in RD patients residing in Tuscany was 85.4%, a higher estimate than that recorded in the general Italian population [10]. Due to the lack of population-based studies exploring the profile of drug utilization in patients with RDs, we compared most of our results with those reported in the report published by the Italian Medicines Agency, in 2018, on drug use in the general population in Italy.

In a manner that was consistent with the results from the general population in Italy [10], we also observed an increase in the prevalence of use with ageing of RD patients aged 20 years and older, with a maximum of 99.4% in patients aged between 80 and 84 years. In adult RD patients, the medications in Groups A and J displayed the highest prevalence in the study period (about 60% for both groups in 2018).

Furthermore, the medications belonging to Groups C, N and A were characterized by the most elevated intensity of use in the adult patients (13.8, 12.9 and 10.5 Pr/Us in 2018, respectively) due to their therapeutic indications for chronic use.

In 2018, drug consumption in adult patients with RDs was approximately five times higher than in the general adult population in Italy (1020 DDD/1000 cases/day), with estimates ranging from 2262.2 in the 20–24 age group to 9813.8 DDD/1000 cases/day in the 80–84 age group. Therefore, elderly people aged 80–84 years with RDs received nearly 10 medication doses every day of the year, which is three times higher than the consumption in the same age group of the general Italian population [10]. Overall, these results support previous findings indicating that the presence of RDs implies a high treatment burden. Indeed, due to their chronic, debilitating and often life-threatening conditions, patients with RDs have higher hospitalization rates and longer hospital stays than the general population [9,20,21], all factors which potentially require multiple therapies in both hospital and home settings.

The female adult patients with RDs showed a higher level of exposure to medications, in line with the results produced by other population-based studies in the general population [22,23]. Furthermore, as with the general population [23], the patients with RDs had a significantly increased prevalence of use in the female gender in specific age groups (i.e., between 20 and 69 years of age) and in most therapeutic categories. The female RD patients were also characterized by a higher consumption of medications for most of the age groups, even among women between 80 and 84 years of age, unlike the general Italian population, for which a more elevated consumption of medications was found in male subjects aged 50 years and older [10]. These differences might suggest the general presence of more severe clinical conditions in females affected by RDs than males, which highlights the tendency of women to receive more days of therapy. On the other hand, in the Italian general population, men reported serious chronic pathologies more frequently than women, implying a longer treatment duration [24].

For the pediatric RD patients, the prevalence of drug use was equal to 71.8% in 2018 (vs. 49.1% in the general pediatric population; [10]), with a maximum reached in the 0–4 age group, indicating the need for high levels of early pediatric drug treatment for patients with RD. Consistently, the prevalence estimates by therapeutic category in 2018 confirmed higher estimates in pediatric patients with RDs compared to the general pediatric population. This was the case in Group J, which showed the greatest prevalence of use (48.2% vs. 38.7%), Group A (23.4% vs. 3.3%), Group H (21.1% vs. 7.5%) and Group R (20.4% vs. 7.0%). In the same year, examining the prevalence estimates of specific substances, the amoxicillin/clavulanic acid combination was the most prescribed product in Group J, with values of prevalence and prescription per 1000 cases of 31.1% and 715.5, respectively, which were almost double those observed in the general population [10], but were comparable with the estimate recorded for the children 0–18 years of age residing in a city in Southern Italy [25]. Another example is provided by the consumption of somatropin (582.9 vs. 15.9 prescriptions per 1000 cases in RD patients and in the Italian general population, respectively) [10], which may be attributable to specific rare conditions such as Turner syndrome, Prader–Willi syndrome, Noonan syndrome and Russell–Silver syndrome, which together represented nearly 30% of the pediatric patients who received at least one prescription of a drug for the nervous system, and in whom long-term growth-hormone treatment has been associated with significant improvements in height and body composition [26,27,28,29]. The highest value for consumption was found in Group N, probably as a consequence of the massive use of antiepileptics, particularly valproic acid, for which there were over 1000 prescriptions per 1000 cases (vs. 52.4 prescriptions per 1000 inhabitants in the national pediatric population in 2018) (Italian Medicines Agency, 2019). Valproic acid is a broad-spectrum antiepileptic drug, effective against all seizure types [30]. In our cohort, in 2018, it was prescribed for 209 patients, representing 6% of the prevalent cases that year and mostly used to treat children with West syndrome, Dravet syndrome, Angelman syndrome and tuberous sclerosis, according to current guidelines [31,32,33,34,35].

Regarding drug consumption in pediatric patients, compared to the general pediatric population, which showed an almost steady trend in drug consumption [10], the children with RDs had the highest value of DDD/1000 cases per day in the 0–4-years-old group (with a maximum reached during the first year of life).

In pediatric RD patients, the prevalence of drug use was significantly higher in males than in females, especially in infants aged 0 to 4 years, in contrast to the lack of appreciable differences found in the general pediatric population in 2018 [10,34]. The consumption of drugs was also higher among male children than in females, except for RD patients between 15 and 19 years; a similar profile was reported for Italian children [10]. In particular, a significant male preponderance was observed in the prevalence of respiratory drugs and hormonal preparations (the most prescribed therapeutic categories in pediatric RD patients), in agreement with previously published findings reporting the higher frequency of their use among boys in general pediatric populations [10,25,36]. Although there are no apparent pharmacological requirements or medical risks related to sex, potential explanations could be due to the different therapeutic indications or to the higher frequency of specific childhood diseases in males compared to females [10].

Overall, these results, in addition to providing an overview of drug use in RD patients, in light of the rational use of medicines advocated by the World Health Organization, may be useful as information for initiating an effective intervention program, such as training, for the promotion of rational drug use and improving the prescription pattern of drugs and the quality of prescriptions [37,38].

### Strengths and Limitations

To the best of our knowledge, this is the first population-based study aimed at evaluating the profile of drug utilization in patients with RDs. The approach applied in this research, based on the linkage of cases between a population-based registry and regional drug-prescription databases, allowed both the recovery of information on almost all the patients diagnosed with RDs in a region of approximately 3.7 million inhabitants and the retrieval of data about pharmaceutical prescriptions and therapies routinely collected at the regional level, thus representing an important tool of public health surveillance. Furthermore, in addition to including RD patients surveilled by the registry from 2000 onwards, this study covers a 10-year period, long enough to provide accurate estimates of the prevalence, intensity of use and consumption of medications in these subjects.

This study also has some limitations. First, only the diseases reported on the list of the Italian law (Decree of the President of the Council of Ministers, 01/2017) are monitored by the Registry of Rare Diseases of Tuscany. For this reason, all the indicators calculated in this paper can be affected by the nature of the RDs represented in our cohort. Thus, our results for the drug utilization of patients with RDs are comparable only to those of studies involving the same group of RDs. Second, given the exclusion of drugs primarily administered in inpatient settings from the analyses, the use of some medications could have been underestimated.

## 5. Conclusions

In this population-based study, the profile of drug utilization was explored in a cohort of patients with RDs residing in a defined geographical area. As in the general population, the prevalence of use and the drug consumption increased with the ageing of the adult RD patients, while in the children, both indicators peaked between 0 and 4 years of age. On the other hand, the presence of these conditions leads to a high burden of drug treatment, as confirmed by the early and greater consumption of drugs compared to the general population. In the years 2008–2018, drugs for the alimentary tract and metabolism in adults and antiinfectives for systemic use in both the adults and the children displayed the highest prevalence of use, while drugs of the nervous system had the highest intensity of use (prescriptions/users) in the pediatric RD patients. Regarding sex differences, the adult patients with RDs exhibited a female preponderance in the prevalence of drug use and consumption in almost all the age groups and therapeutic categories; conversely, a higher prevalence of use was found in the male pediatric population.

Collectively, these findings provide relevant information on the drug profile of patients with RDs and also represent a first step for future analyses to monitor drug utilization and changes in the profile of the use of therapeutic categories in RD patients over time. Furthermore, these results suggest the importance of the correct epidemiological monitoring of drug prescriptions in order to increase the rational use of medicines in this category of patients.

## Figures and Tables

**Table 1:** Anatomical therapeutic chemical classification system.

Abbreviation	Category Name
A	Alimentary tract and metabolism
B	Blood and blood-forming organs
C	Cardiovascular system
D	Dermatologicals
G	Genito-urinary system and sex hormones
H	Systemic hormonal preparations, excl. sex hormones and insulin
J	Antiinfectives for systemic use
L	Antineoplastic and immunomodulating agents
M	Musculo-skeletal system
N	Nervous system
P	Antiparasitic products, insecticides and repellents
R	Respiratory system
S	Sensory organs
V	Various *

* including the following subgroups: V01—allergen extracts; V03—all other therapeutic products; V04—diagnostic agents; V06—general nutrients; V07—all other non-therapeutic products; V08—contrast media.

**Table 2:** The most prescribed substances for RD patients in pediatric age by therapeutic category in the last year of the study period (2018).

ATC	Therapeutic Category/Substance	Prevalence (%)	Prescriptions (Per 1000 Cases)
J	Antiinfectives for systemic use	48.2	2004
J01CR02	Amoxicillin/clavulanic acid	31.2	715
J01DD08	Cefixime	9.4	213
J01FA10	Azithromycin	6.5	201
J01FA09	Clarithromycin	5.5	90
J01CA04	Amoxicillin	5.1	147
A	Alimentary tract and metabolism	23.4	2116
A11CC05	Cholecalciferol	12.0	384
A02BC03	Lansoprazole	3.5	319
A05AA02	Ursodeoxycholic acid	1.2	108
A02BC05	Esomeprazole	1.1	124
H	Systemic hormonal preparations, excl. sex hormones and insulins	21.1	1164
H02AB01	Betamethasone	12.5	248
H01AC01	Somatropin	3.7	583
H03AA01	Levothyroxine	2.3	129
H02AB07	Prednisone	1.7	84
H01BA02	Desmopressin	0.5	26
R	Respiratory system	20.4	762
R03AC02	Salbutamol	8.2	193
R03BA01	Beclomethasone	6.2	113
R03BA05	Fluticasone	4.6	125
R03BA02	Budesonide	3.7	71
R06AE07	Cetirizine	2.5	54
R03DC03	Montelukast	1.0	54
N	Nervous System	13.5	2827
N03AG01	Valproic acid	5.5	1003
N03AF01	Carbamazepine	1.8	199
N03AX14	Levetiracetam	0.9	92

## Data Availability

The data supporting the findings of this study are available from Regione Toscana but restrictions apply to the availability of these data, which were used under license for the current study, and therefore are not publicly available. Data are however available from the authors upon reasonable request and with permission of Regione Toscana. Requests to access the datasets should be directed to Regione Toscana, https://www.regione.toscana.it/.

## Supplementary Materials

The following supporting information can be downloaded at: https://www.mdpi.com/article/10.3390/ijerph20020937/s1, Table S1. List of rare diseases endowed of an exemption code as defined by the Italian law and included in the study. For each nosological group, groups of diseases are reported in bold and, if available, specific diseases belonging to the groups are indicated.
