# Supplementary material for: Profile of Drug Utilization in Patients with Rare Diseases in Tuscany, Italy: A Population-Based Study
Source: Int J Environ Res Public Health. 2023 Jan 4;20(2):937. doi: 10.3390/ijerph20020937 (PMC9858964; doi:10.3390/ijerph20020937)
Supplement: Supplementary file 1 [file ijerph-20-00937-s001.zip › ijerph-2096646-supplementary.pdf]

Table S1. List of rare diseases endowed of an exemption code as defined by the Italian law and included in the study. For each nosological group, groups of diseases are reported in bold and, if available, specific diseases belonging to the groups are indicated.

**INFECTIOUS AND PARASITIC DISEASES**	
Disease and/or group	*Specific diseases included in the group*
LEPROSY	
LYME DISEASE	
WHIPPLE DISEASE	
**NEOPLASMS**	
Disease and/or group	*Specific diseases included in the group*
**CARNEY COMPLEX**	
FAMILIAL ADENOMATOUS POLYPOSIS	
GARDNER SYNDROME	
GORLIN SYNDROME RETINOBLASTOMA	
**HEREDITARY NONPOLYPOSIS COLON CANCER**	Lynch syndrome
LYMPHANGIOLEIOMYOMATOSIS	
NEUROFIBROMATOSIS	
NEPHROBLASTOMA	
RETINOBLASTOMA	
**ENDOCRINE DISEASES**	
Disease and/or group	*Specific diseases included in the group*
**ADRENOGENITAL SYNDROME**	
**AUTOIMMUNE POLYENDOCRINOPATHY**	Schmidt's syndrome
CONGENITAL HYPOGONADOTROPIC HYPOGONADISM	
CONGENITAL ISOLATED ACTH DEFICIENCY	
FAMILIAL MEDULLARY THYROID CARCINOMA	
IDIOPATHIC CENTRAL PRECOCIOUS PUBERTY	
KALLMANN SYNDROME	
LARON SYNDROME	
LEPRECHAUNISM	
MULTIPLE ENDOCRINE NEOPLASIA TYPE 1	
MULTIPLE ENDOCRINE NEOPLASIA TYPE 2A	

NON-ACQUIRED ISOLATED GROWTH HORMONE DEFICIENCY	
PENDRED SYNDROME	
REFETOFF SYNDROME	
**RARE PRIMARY HYPERALDOSTERONISM**	Primary hyperaldosteronism-seizures-neurological abnormalities syndrome<br>Primary unilateral adrenal hyperplasia
**METABOLIC DISEASES**	
Disease and/or group	*Specific diseases included in the group*
**DISORDERS OF MITOCHONDRIAL METABOLISM**	
**CREATINE DEFICIENCY SYNDROME**	
KEARNS-SAYRE SYNDROME	
**ISOLATED OXIDATIVE PHOSPHORYLATION COMPLEX DISORDER**	
LEIGH SYNDROME	
LEBER OPTIC ATROPHY	
LONG CHAIN 3-HYDROXYACYL-COA DEHYDROGENASE DEFICIENCY	
MEDIUM CHAIN ACYL-COA DEHYDROGENASE DEFICIENCY	
MELAS	
MERFF	
**MITOCHONDRIAL OXIDATIVE PHOSPHORYLATION DISORDER DUE TO MITOCHONDRIAL DNA ANOMALIES**	
**MITOCHONDRIAL OXIDATIVE PHOSPHORYLATION DISORDER DUE TO NUCLEAR DNA ANOMALIES**	
**OTHER DISORDER OF MITOCHONDRIAL METABOLISM**	Epileptic encephalopathy with global cerebral demyelination
PYRUVATE DEHYDROGENASE PHOSPHATASE DEFICIENCY	
SHORT CHAIN ACYL-COA DEHYDROGENASE DEFICIENCY	
VERY LONG CHAIN ACYL-COA DEHYDROGENASE DEFICIENCY	
**LYSOSOMAL STORAGE DISEASES**	
GALACTOSIALIDOSIS	
**GANGLIOSIDOSIS**	
KRABBE DISEASE	

**LIPID STORAGE DISEASE**	Fabry disease<br>Gaucher disease<br>Niemann-Pick disease<br>Niemann-Pick disease type C
METACHROMATIC LEUKODYSTROPHY	
MUCOLIPIDOSIS	
**MUCOPOLYSACCHARIDOSIS**	Mucopolysaccharidosis type 4
**NEURONAL CEROID LIPOFUSCINOSIS**	
**OTHER LYSOSOMAL STORAGE DISEASES**	Glycogen storage disease due to LAMP-2 deficiency
**DISORDERS OF VITAMIN AND NON-PROTEIN COFACTOR ABSORPTION AND TRANSPORT**	
BIOTINIDASE DEFICIENCY	
**DISORDER OF COBALAMIN AND FOLATE METABOLISM AND TRANSPORT**	Methylmalonic acidemia with homocystinuria, type cblC
**DISORDER OF OTHER VITAMINS AND COFACTORS METABOLISM AND TRANSPORT***<br>*excludes Ataxia with vitamin E deficiency	
HYPOCALCEMIC VITAMIN D-DEPENDENT RICKETS	
HYPOPHOSPHATEMIC RICKETS	
**DISORDERS OF METAL METABOLISM AND TRANSPORT**	
ACERULOPLASMINEMIA	
**DISORDER OF IRON METABOLISM AND TRANSPORT**	Rare hereditary hemochromatosis<br>Hereditary hyperferritinemia-cataract syndrome
MENKES DISEASE	
WILSON DISEASE	
**DISORDERS OF PROTEIN METABOLISM AND TRANSPORT**	
**CONGENITAL DISORDER OF GLYCOSYLATION**	
CRIGLER-NAJJAR SYNDROME	
**PRIMARY SYSTEMIC AMYLOIDOSIS**	Wild type ATTR amyloidosis
**OTHER METABOLIC DISEASES**	
ADIPOSIS DOLOROSA	

ADRENOLEUKODYSTROPHY	
CEREBROTENDINOUS XANTHOMATOSIS	
**DISORDER OF AMINO ACID ABSORPTION AND TRANSPORT**	Albinism<br>Alkaptonuria<br>Cystinosis<br>Cystinuria<br>Glutaric aciduria<br>Glycine encephalopathy<br>Hyperprolinemia<br>Homocystinuria<br>Maple syrup urine disease<br>Methylmalonic acidemia<br>Phenylketonuria/ Hyperphenylalaninemia due to tetrahydrobiopterin deficiency<br>Pyruvate dehydrogenase E1-alpha deficiency<br>Tyrosinemia<br>Other acidemias or primitive organic acidurias from metabolism defects of branched chain amino acids
**DISORDER OF CARBOHYDRATE METABOLISM***<br>*excludes Diabetes mellitus	Fructose-1,6-bisphosphatase deficiency<br>Glucose transport disorder<br>Galactosemia<br>Glycogen storage disease<br>Glycogen storage disease due to acid maltase deficiency<br>Hereditary fructose intolerance
**DISORDER OF LIPID METABOLISM***<br>*excludes Combined familial hyperlipidemia, Dysbetalipoproteinemia, Heterozigous familial hypercholesterolemia type IIA and IIB, Polygenic hypercholesterolemia	Familial lipoprotein lipase deficiency<br>Homozygous familial hypercholesterolemia<br>Familial chylomicronemia syndrome<br>Hypobetalipoproteinemia<br>Tangier disease
DISORDER OF NEUROTRANSMITTER AND PEPTIDE METABOLISM	
**DISORDER OF PURINE OR PYRIMIDINE METABOLISM**	Lesch-Nyhan syndrome
**DISORDER OF UREA CYCLE METABOLISM AND AMMONIA DETOXIFICATION**	Citrullinemia<br>Ornithine transcarbamylase deficiency

FAMILIAL TUMORAL CALCINOSIS	
**FAMILIAL HYPERINSULINISM**	
GENERALIZED LIPODYSTROPHY	
HYPOPHOSPHATASIA	
**PEROXISOMAL DISEASE**	
PORPHYRIA	
REFSUM DISEASE	
ZELLWEGER SYNDROME	
**IMMUNE SYSTEM DISORDERS**	
Disease and/or group	*Specific diseases included in the group*
ACQUIRED ANGIOEDEMA WITH C1INH DEFICIENCY	
ALPHA-1-ANTITRYPSIN DEFICIENCY	
**AUTOINFLAMMATORY SYNDROME**	Periodic fever syndrome<br>Hyperimmunoglobulinemia D with periodic fever<br>Cryopyrin-associated periodic syndrome
**CHRONIC HISTIOCYTOSIS**	Erdheim-Chester disease<br>Langerhans cell histiocytosis<br>Histiocytosis X
FAMILIAL MEDITERRANEAN FEVER	
HEREDITARY ANGIOEDEMA	
NEONATAL ANTIPHOSPHOLIPID SYNDROME	
**PRIMARY IMMUNODEFICIENCY**	Agammaglobulinemia<br>DiGeorge syndrome
SCHNITZLER SYNDROME	
TUMOR NECROSIS FACTOR RECEPTOR 1 ASSOCIATED PERIODIC SYNDROME	
**DISEASES OF THE BLOOD AND BLOOD-FORMING ORGANS**	
Disease and/or group	*Specific diseases included in the group*
CYCLIC NEUTROPENIA	
CHRONIC GRANULOMATOUS DISEASE	
**CHRONIC PRIMARY PLATELET DISORDER**	Immune thrombocytopenia
HEMOLYTIC UREMIC SYNDROME	

**HEREDITARY ANEMIA***<br>*excludes Class I glucose-6-phosphate dehydrogenase deficiency	Blackfan-Diamond anemia<br>Fanconi anemia<br>Hereditary spherocytosis<br>Sickle cell anemia<br>Sideroblastic anemia<br>Thalassemia
**INHERITED COAGULATION DISORDER**	Hemophilia A<br>Hemophilia B<br>Rare hemorrhagic disorder due to a constitutional coagulation factors defect<br>Rare hereditary thrombophilia<br>Von Willebrand disease
**MYELODYSPLASTIC SYNDROME**	Refractory anemia
PAROXYSMAL NOCTURNAL HEMOGLOBINURIA	
RARE ACQUIRED APLASTIC ANEMIA*<br>*excludes Transitory medullary aplasia	
**RARE HEMORRHAGIC DISORDER DUE TO A CONSTITUTIONAL PLATELET ANOMALY**	
**RARE HEMORRHAGIC DISORDER DUE TO A CONSTITUTIONAL THROMBOCYTOPENIA**	
**SEVERE CONGENITAL NEUTROPENIA**	Adult idiopathic neutropenia
SHWACHMAN-DIAMOND SYNDROME	
SYSTEMIC MASTOCYTOSIS	
**PERIPHERAL AND CENTRAL NERVOUS SYSTEM DISORDERS**	
Disease and/or group	*Specific diseases included in the group*
AMYOTROPHIC LATERAL SCLEROSIS	
**AUTOSOMAL DOMINANT CEREBELLAR ATAXIA**	Ataxia with vitamin E deficiency<br>Cerebellar ataxia-hypogonadism syndrome<br>Fragile X-associated tremor/ataxia syndrome<br>Friedreich ataxia<br>Hereditary episodic ataxia<br>Hereditary spastic paraplegia<br>Marie's cerebellar ataxia<br>Marinesco-Sjögren syndrome

	Pantothenate kinase-associated neurodegeneration
BILATERAL STRIOPALLIDODENTATE CALCINOSIS	
CEREBRAL AUTOSOMAL DOMINANT ARTERIOPATHY-SUBCORTICAL INFARCTS-LEUKOENCEPHALOPATHY	
CHRONIC INFLAMMATORY DEMYELINATING POLYNEUROPATHY	
**CONGENITAL MYASTHENIC SYNDROME**	Myasthenia gravis
**CONGENITAL MYOPATHY**	Central core disease<br>Centronuclear myopathy<br>Nemaline myopathy<br>Qualitative or quantitative defects of desmin
DENTATORUBRAL PALLIDOLUYSIAN ATROPHY	
DRAVET SYNDROME	
FAMILIAL OR SPORADIC HEMIPLEGIC MIGRAINE	
**GENETIC PERIPHERAL NEUROPATHY**	Charcot-Marie-Tooth disease/Hereditary motor and sensory neuropathy<br>Dejerine-Sottas syndrome<br>Giant axonal neuropathy<br>Hereditary neuropathy with liability to pressure palsies<br>Hereditary sensory and autonomic neuropathy<br>Roussy-Lévy syndrome
GUILLAIN-BARRÉ SYNDROME	
HUNTINGTON DISEASE	
IDIOPATHIC TORSION DYSTHONIA	
ISAAC SYNDROME	
LAMBERT-EATON MYASTHENIC SYNDROME	
LANDAU-KLEFFNER SYNDROME	
LENNOX-GASTAUT SYNDROME	
**LEUKODYSTROPHY**	Aicardi-Goutières syndrome<br>Alexander disease<br>Pelizaeus-Merzbacher disease
MELKERSSON-ROSENTHAL SYNDROME	
MULTIFOCAL MOTOR NEUROPATHY	
MULTIPLE SYSTEM ATROPHY	

**MUSCULAR DYSTROPHY**	Becker muscular dystrophy<br>Calpain-3-related limb-girdle muscular dystrophy R1<br>Duchenne muscular dystrophy<br>Facioscapulohumeral dystrophy<br>Oculogastrointestinal muscular dystrophy
**MYOTONIC DYSTROPHY**	Paramyotonia congenita of Von Eulenburg<br>Steinert myotonic dystrophy<br>Thomsen and Becker disease
NARCOLEPSY	
**NEURODEGENERATION WITH BRAIN IRON ACCUMULATION**	Infantile neuroaxonal dystrophy
NEURONAL INTRANUCLEAR INCLUSION DISEASE	
**PERIODIC PARALYSIS**	
POEMS SYNDROME	
**PRIMARY DYSTONIA**	
PRIMARY LATERAL SCLEROSIS	
PROGRESSIVE MYOCLONIC EPILEPSY	
PROGRESSIVE SUPRANUCLEAR PALSY	
RETT SYNDROME	
RIBOFLAVIN TRANSPORTER DEFICIENCY	
**SPINAL MUSCULAR ATROPHY**	Kennedy disease<br>Proximal spinal muscular atrophy type 1<br>Proximal spinal muscular atrophy type 3
STIFF PERSON SPECTRUM DISORDER	
SYRINGOMYELIA	
WEST SYNDROME	
**DISORDERS OF THE EYE AND ADNEXA**	
Disease and/or group	*Specific diseases included in the group*
COGAN SYNDROME	
CONGENITAL STATIONARY NIGHT BLINDNESS	
**CORNEAL DYSTROPHY**	Epithelial basement membrane dystrophy<br>Fuchs endothelial corneal dystrophy<br>Granular corneal dystrophy type II

	Macular corneal dystrophy<br>Meesmann corneal dystrophy<br>Posterior polymorphous corneal dystrophy<br>Reis-Bücklers corneal dystrophy
FAMILIAL EXUDATIVE VITREORETINOPATHY	
**ISOLATED CHORIORETINAL DYSTROPHY**	
**ISOLATED INHERITED RETINAL DISORDER**	Best vitelliform macular dystrophy<br>Leber congenital amaurosis<br>Progressive cone dystrophy<br>Retinitis pigmentosa<br>Retinitis punctata albescens<br>Stargardt disease<br>Vitreoretinal distrophy
OGUCHI DISEASE	
POSTERIOR UVEITIS	
SERPIGINOUS CHOROIDITIS	
SYNDROMIC KERATOCONUS	
**CIRCULATORY SYSTEM DISEASES**	
Disease and/or group	*Specific diseases included in the group*
ANTI-GLOMERULAR BASEMENT MEMBRANE DISEASE	
BEHÇET DISEASE	
BUDD-CHIARI SYNDROME	
CRYOGLOBULINEMIC VASCULITIS	
EOSINOPHILIC GRANULOMATOSIS WITH POLYANGIITIS	
GIANT CELL ARTERITIS	
GRANULOMATOSIS WITH POLYANGIITIS	
HEREDITARY HEMORRHAGIC TELANGIECTASIA	
IMMUNOGLOBULIN A VASCULITIS	
MICROSCOPIC POLYANGIITIS	
POLYARTERITIS NODOSA	
**PRIMARY LYMPHEDEMA**	Milroy disease<br>Meige disease<br>Idiopathic primary lymphedema

	Recessive primary congenital lymphedema
RHEUMATIC HEART DISEASE	
TAKAYASU ARTERITIS	
**THROMBOTIC MICROANGIOPATHY**	Hemolytic uremic syndrome<br>Thrombotic thrombocytopenic purpura
**RESPIRATORY DISEASES**	
Disease and/or group	*Specific diseases included in the group*
AUTOIMMUNE PULMONARY ALVEOLAR PROTEINOSIS	
IDIOPATHIC PULMONARY ARTERIAL HYPERTENSION	
IDIOPATHIC PULMONARY HEMOSIDEROSIS	
ONDINE SYNDROME	
PRIMARY CILIARY DYSKINESIA	
PRIMARY CILIARY DYSKINESIA, KARTAGENER TYPE	
**PRIMARY INTERSTITIAL LUNG DISEASE IN CHILDHOOD AND ADULTHOOD**	Acute interstitial pneumonia<br>Idiopathic pulmonary fibrosis
SARCOIDOSIS	
**DIGESTIVE DISORDERS**	
Disease and/or group	*Specific diseases included in the group*
CHRONIC INTESTINAL PSEUDOOBSTRUCTION	
CONGENITAL CHLORIDE DIARRHEA	
EOSINOPHILIC GASTROENTERITIS	
**ISOLATED ACHALASIA AND SYNDROMES-ASSOCIATED ACHALASIA**	Triple A syndrome
PRIMARY INTESTINAL LYMPHANGIECTASIA	
PRIMARY SCLEROSING CHOLANGITIS	
**PROGRESSIVE FAMILIAL INTRAHEPATIC CHOLESTASIS**	Progressive familial intrahepatic cholestasis type 3
**DISEASES OF THE GENITOURINARY SYSTEM**	
Disease and/or group	*Specific diseases included in the group*
ALPORT SYNDROME	
IGG4-RELATED RETROPERITONEAL FIBROSIS	
INTERSTITIAL CYSTITIS	

NEPHROGENIC DIABETES INSIPIDUS	
**PRIMARY GLOMERULAR DISEASE**	
**RARE RENAL TUBULAR DISEASE**	Bartter syndrome<br>Dent disease<br>Gitelman syndrome
**DISEASES OF THE SKIN AND SUBCUTANEOUS TISSUE**	
Disease and/or group	*Specific diseases included in the group*
APLASIA CUTIS CONGENITA	
BULLOUS PEMPHIGOID	
CONGENITAL NON-BULLOUS ICHTHYOSIFORM ERYTHRODERMA	
CUTIS LAXA	
DARIER DISEASE	
DIFFUSE CUTANEOUS SYSTEMIC SCLEROSIS	
DYSKERATOSIS CONGENITA	
EEC SYNDROME	
**ECTODERMAL DYSPLASIA SYNDROME**	
EPIDERMAL NEVUS SYNDROME	
ERYTHROKERATODERMIA VARIABILIS	
FAMILIAL ATYPICAL MULTIPLE MOLE MELANOMA SYNDROME	
**HEREDITARY PALMOPLANTAR KERATODERMA**	
HYPOMELANOSIS OF ITO	
IBIDS SYNDROME	
INCONTINENTIA PIGMENTI	
INHERITED EPIDERMOLYSIS BULLOSA	
**INHERITED ICHTHYOSIS**	Autosomal recessive congenital ichthyosis<br>Lamellar ichthyosis<br>Netherton syndrome<br>X-linked ichthyosis
KERATOSIS FOLLICULARIS SPINULOSA DECALVANS	
LICHEN SCLEROSUS ET ATROPHICUS	
MUCOUS MEMBRANE PEMPHIGOID	
PEMPHIGUS	
PSEUDOXANTHOMA ELASTICUM	

PYODERMA GANGRENOSUM	
SJÖGREN-LARSSON SYNDROME	
**DISEASES OF THE MUSCULOSKELETAL SYSTEM AND CONNECTIVE TISSUE**	
Disease and/or group	*Specific diseases included in the group*
ANTISYNTHETASE SYNDROME	
DERMATOMYOSITIS	
DIFFUSE FASCIITIS	
EOSINOPHILIC FASCIITIS	
FIBRODYSPLASIA OSSIFICANS PROGRESSIVA	
GORHAM-STOUT DISEASE	
INCLUSION BODY MYOSITIS	
MIXED CONNECTIVE TISSUE DISEASE	
POLYMYOSITIS	
RELAPSING POLYCHONDRITIS	
SAPHO SYNDROME	
SYSTEMIC SCLEROSIS	
**CONGENITAL ANOMALIES, CHROMOSOMAL ABERRATIONS AND GENETIC SYNDROMES**	
Disease and/or group	*Specific diseases included in the group*
**CONGENITAL MALFORMATIONS OF THE NERVOUS SYSTEM**	
ARNOLD-CHIARI MALFORMATION	
CEREBELLAR AGENESIS	
GERSTMANN SYNDROME	
ISOLATED OR SYNDROMIC HOLOPROSENCEPHALY	
ISOLATED OR SYNDROMIC MICROCEPHALY	
JOUBERT SYNDROME AND RELATED DISORDERS	
LISSENCEPHALY	
**SYNDROME WITH CORPUS CALLOSUM AGENESIS/DYSGENESIS AS A MAJOR FEATURE**	Dandy-Walker sydrome
WALKER-WARBURG SYNDROME	
**OTHER SEVERE AND DISABLING SYNDROMES WITH A CENTRAL NERVOUS SYSTEM MALFORMATION AS A MAJOR FEATURE**	

**CONGENITAL MALFORMATIONS OF THE EYE**	
ANIRIDIA	
AXENFELD-RIEGER SYNDROME	
COLOBOMA OF THE OPTIC NERVE	
**ISOLATED OR SYNDROMIC CONGENITAL OCULAR COLOBOMA**	
**MICROPHTHALMIA-ANOPHTHALMIA-COLOBOMA**	Microphthalmia, Lenz type
PETERS ANOMALY	
RIEGER ANOMALY	
SEPTO-OPTIC DYSPLASIA SPECTRUM	
VOGT-KOYANAGI-HARADA DISEASE	
**ISOLATED OR SYNDROMIC CONGENITAL CRANIOFACIAL ANOMALIES**	
ACROCEPHALOSYNDACTYLY	
ANTLEY-BIXLER SYNDROME	
APERT SYNDROME	
PFEIFFER SYNDROME	
PIERRE ROBIN SYNDROME	
**OTHER SEVERE AND DISABLING ANOMALIES OF SKULL AND FACE BONES, INTEGUMENTS AND MUCOSA**	Crouzon disease<br>Frontofacionasal dysplasia<br>Isolated craniosynostosis<br>Isolated or syndromic cleft palate
**CONGENITAL FACIAL ABNORMALITIES**	
GOLDENHAR SYNDROME	
MOEBIUS SYNDROME	
SCHINZEL-GIEDION SYNDROME	
**CONGENITAL LIMB MALFORMATIONS**	
ADAMS-OLIVER SYNDROME	
**ARTHROGRYPOSIS MULTIPLEX CONGENITA**	
CONGENITAL ABSENCE OF UPPER ARM AND FOREARM WITH HAND PRESENT	
FEMORAL-FACIAL SYNDROME	
FREEMAN-SHELDON SYNDROME	

POLAND SYNDROME	
**CONGENITAL HEART DISEASE**	
KLIPPEL-TRÉNAUNAY SYNDROME	
**RARE CONGENITAL NON-SYNDROMIC HEART MALFORMATION***<br>*excludes Isolated atrial septal defect, Isolated pulmonary valve stenosis, Isolated ventricular septal defect, Patent ductus arteriosus	Ebstein malformation of the tricuspid valve<br>Hypoplastic left heart syndrome
**OTHER SEVERE AND DISABLING CONGENITAL SYNDROMIC VASCULAR DISEASE**	CLOVES syndrome<br>Capillary malformation-arteriovenous malformation
**ABDOMINAL WALL DEFECTS**	
ISOLATED KLIPPEL-FEIL SYNDROME	
**CONGENITAL ANOMALIES OF THE GASTROINTESTINAL TRACT**	
ANORECTAL MALFORMATION	
CAROLI DISEASE	
DUPLICATION OF THE GASTROINTESTINAL TRACT	
ESOPHAGEAL ATRESIA AND/OR ISOLATED TRACHEOESOPHAGEAL FISTULA	
ISOLATED BILIARY ATRESIA	
ISOLATED POLYCYSTIC LIVER DISEASE	
HIRSCHSPRUNG DISEASE	
PERSISTENT CLOACA	
**CONGENITAL UROGENITAL ANOMALIES**	
AUTOSOMAL RECESSIVE POLYCYSTIC KIDNEY DISEASE	
BLADDER EXSTROPHY	
**DISORDER OF SEXUAL DEVELOPMENT AND/OR AMBIGUOUS GENITALIA AND/OR KARIOTYPE DISCORDANCE/GONADAL DEVELOPMENT AND/OR PHENOTYPE**	Gonadal dysgenesis<br>Complete androgen insensitivity syndrome<br>Partial androgen insensitivity syndrome
MAYER-ROKITANSKY-KÜSTER-HAUSER SYNDROME	
**FAMILIAL CYSTIC RENAL DISEASE**	
MEDULLARY SPONGE KIDNEY	

**PSEUDOHERMAPHRODITISM**	
**OTHER SEVERE AND DISABLING DEFECTS OF SEXUAL DEVELOPMENT AND/OR AMBIGUOUS GENITALIA AND/OR KARIOTYPE DISCORDANCE/GONADAL DEVELOPMENT AND/OR PHENOTYPE**	
**GENETIC SKELETAL DISORDERS**	
ACRODYSOSTOSIS	
**CONGENITAL CHONDRODISPLASIAS**	Achondroplasia<br>Jeune syndrome<br>Multiple osteochondromas
**CONGENITAL ISOLATED OR SYNDROMIC CHONDRODISPLASIAS**	Camurati-Engelmann disease<br>Craniometaphyseal dysplasia<br>Fibrous dysplasia<br>Léri-Weill dyschondrosteosis<br>Multiple epiphyseal dysplasia<br>McCune-Albright syndrome<br>Osteogenesis imperfecta<br>Osteopetrosis and related disorders<br>Spondyloepiphyseal dysplasia tarda
MAFFUCCI SYNDROME	
SPONDYLOEPIPHYSEAL DYSPLASIA CONGENITA	
**OTHER SYNDROMES AND COMPLEX CONGENITAL MALFORMATIONS**	
22q11.2 DELETION SYNDROME	
AARSKOG-SCOTT SYNDROME	
ALAGILLE SYNDROME	
ALSTRÖM SYNDROME	
ANGELMAN SYNDROME	
BARDET-BIEDL SYNDROME	
BECKWITH-WIEDEMANN SYNDROME	
BOR SYNDROME	
BORJESON-FORSSMAN-LEHMANN SYNDROME	
BRANCHIO-OCULO-FACIAL SYNDROME	

CARDIOFACIOCUTANEOUS SYNDROME	
CHAR SYNDROME	
CHARGE SYNDROME	
COCKAYNE SYNDROME	
COFFIN-SIRIS SYNDROME	
COHEN SYNDROME	
CORNELIA DE LANGE SYNDROME	
DUBOWITZ SYNDROME	
EHLERS-DANLOS SYNDROME	
FRAGILE X SYNDROME	
HOLT-ORAM SYNDROME	
ISOLATED HEMIHYPERPLASIA	
KABUKI SYNDROME	
LOEYS-DIETZ SYNDROME	
MARFAN SYNDROME	
MARSHALL SYNDROME	
MARSHALL-SMITH SYNDROME	
MONOSOMY 5P	
**MOSAIC VARIEGATED ANEUPLOIDY SYNDROME**	
**MULTIPLE CONGENITAL ANOMALIES/DYSMORPHIC SYNDROME-INTELLECTUAL DISABILITY**	KBG syndrome
**MULTIPLE HAMARTOMAS**	Cowden syndrome<br>Hepatic cystic hamartoma
NAIL-PATELLA SYNDROME	
NOONAN SYNDROME AND NOONAN-RELATED SYNDROME	
NOONAN SYNDROME WITH MULTIPLE LENTIGINES	
OCULODENTODIGITAL DYSPLASIA	
OPITZ GBBB SYNDROME	
PEUTZ-JEGHERS SYNDROME	
PRADER-WILLI SYNDROME	
PROGRESSIVE HEMIFACIAL ATROPHY	
RUBINSTEIN-TAYBI SYNDROME	
SALDINO-MAINZER SYNDROME	
SECKEL SYNDROME	

SILVER-RUSSELL SYNDROME	
SMITH-MAGENIS SYNDROME	
SOTOS SYNDROME	
STICKLER SYNDROME	
STURGE-WEBER SYNDROME	
**SYNDROMIC CHROMOSOMAL AND GENOMIC UNBALANCED REARRANGEMENTS**	Distal 22q11.2 microdeletion syndrome
TETRASOMY 12P	
TOWNES-BROCKS SYNDROME	
TUBEROUS SCLEROSIS COMPLEX	
TURNER SYNDROME	
VACTERL/VATER ASSOCIATION	
VON HIPPEL-LINDAU DISEASE	
WAGR SYNDROME	
**WAARDENBURG SYNDROME**	
WILLIAMS SYNDROME	
WOLF-HIRSCHHORN SYNDROME	
WOLFRAM SYNDROME	
**NEONATAL MORBITIES OF PERINATAL ORIGIN**	
Disease and/or group	*Specific diseases included in the group*
BILIRUBIN ENCEPHALOPATHY	
FETAL ALCOHOL SYNDROME	
HEPATIC FIBROSIS-RENAL CYSTS-INTELLECTUAL DISABILITY SYNDROME	
